# Longitudinal changes in hamstring muscle morphology in adolescent competitive alpine skiers are sex-specific: a 4-year cohort study

**DOI:** 10.1080/07853890.2026.2620897

**Published:** 2026-02-01

**Authors:** Daniel P. Fitze, Jonas Hanimann, Martino V. Franchi, Stefan Fröhlich, Johannes Scherr, Jess G. Snedeker, Reto Sutter, Jörg Spörri

**Affiliations:** ^a^ Sports Medical Research Group, Department of Orthopaedics, Balgrist University Hospital, University of Zurich, Zurich, Switzerland; ^b^ Department of Orthopaedics, Balgrist University Hospital, University Center for Prevention and Sports Medicine, University of Zurich, Zurich, Switzerland; ^c^ Department of Biomedical Sciences, University of Padua, Padua, Italy; ^d^ Biomechanics Laboratory, Department of Orthopaedics, Balgrist University Hospital, University of Zurich, Zurich, Switzerland; ^e^ Institute for Biomechanics, ETH Zurich, Zurich, Switzerland; ^f^ Department of Radiology, Balgrist University Hospital, University of Zurich, Zurich, Switzerland

**Keywords:** Athletes, biological maturation, injury prevention, muscle architecture, muscle size

## Abstract

**Background:**

The aim of this study was to investigate changes in hamstring architecture and size over a 4-year period, explore sex-specific differences, and examine associations with biological maturation in adolescent competitive alpine skiers.

**Materials and methods:**

59 competitive alpine skiers (27 females, 32 males; baseline age: 14.9 ± 0.7 years) competing at regional to national level were examined at baseline and follow-up. BMI was calculated and biological maturation estimated using the sex-specific Mirwald equation. Ultrasound imaging assessed biceps femoris long head architecture and maximal anatomical cross-sectional area (ACSA_max_) of all hamstring muscles. ACL injuries were retrospectively recorded and verified using medical records and MRI. Participants were grouped as uninjured (*n* = 53) and ACL-injured (*n* = 6).

**Results:**

Mixed ANOVA revealed significant main and interaction effects for hamstring muscle size, but not for architecture. Post hoc analyses showed that hamstring muscle size increased in both sexes (all *p* < 0.001), except for the semitendinosus in females (*p* = 0.499). Biological maturation was associated with changes in femur length (*R*² = 0.32, *p* < 0.001) and total hamstring muscle size (*R*² = 0.52, *p* < 0.001). As a secondary observation, female skiers who later sustained an ACL injury (*n* = 5) descriptively showed smaller semitendinosus size across both time points.

**Conclusions:**

This study provides new insights into hamstring development in adolescent alpine skiers and highlights the role of biological maturation. Hamstring size increased, with greater hypertrophy in males. The smaller semitendinosus size in ACL-injured females may be relevant for injury prevention.

## Introduction

Alpine ski racing is a high-speed sport that is associated with a high risk of injury to the lower extremities [1**–**3]. Among these injuries, the knee joint is most frequently affected [1,3], and injuries to the anterior cruciate ligament (ACL) are particularly common [2]. ACL injuries are especially critical in alpine ski racing due to the long rehabilitation period and the increased risk of re-injury [4]. ACL injuries are often accompanied by simultaneous damage to other knee structures such as the meniscus or articular cartilage, which can accelerate joint degeneration and increase the risk of developing degenerative joint diseases such as knee osteoarthritis [5].

The mechanisms leading to ACL injuries usually involve complex knee dynamics, such as tibial translation relative to the femur, dynamic knee valgus movement and internal tibial rotation [6,7]. These injury mechanisms emphasize the essential role of the hamstring (HAM) muscles, in particular the biceps femoris long head (BFlh), which is capable of acting synergistically with the ACL by counteracting these movements [8]. The BFlh has the ability to generate large posterior shear forces, and thus mitigate the anterior tibial translation and internal rotation often associated with ACL injuries [9].

During growth and maturation, significant changes in muscle morphology occur, including fascicle length (Lf), pennation angle (PA) and maximal anatomical cross-sectional area (ACSA_max_). The review by Kumar and colleagues [10] summarized that moderate to large increases in Lf, PA and muscle size occur during adolescence, with male adolescents experiencing greater hypertrophy adaptations due to hormonal differences. These changes can be muscle- and region-specific and are the result of a complex interaction between biological maturation and training stimulus. Therefore, a more comprehensive understanding of these interactions could be beneficial for the development of maturation-adequate training programmes to enhance performance and to reduce the risk of injury in adolescent athletes.

Previous cross-sectional studies conducted by our research group on competitive alpine skiers have provided important insights into HAM muscle strength and morphology [11–13]. For example, in a cross-sectional study that assessed the maximal eccentric HAM strength during Nordic HAM curls [11], differences were found between adolescent and elite alpine skiers. In adolescent skiers, this strength correlated with biological maturation. In a cohort study involving adolescent alpine skiers [12], U16 skiers displayed longer BFlh Lf than U15 skiers. Furthermore, associations were found between biological maturation and the Lf and the average ACSA of the BFlh, but not between biological maturation and PA. A smaller average BFlh ACSA was also associated with an increased risk of sustaining a traumatic lower extremity injury in the following season. A recent cross-sectional study of elite ski racers established normative reference values for the HAM and quadriceps muscle size and strength. Male skiers demonstrated a larger ACSA_max_ for the BFlh, biceps femoris short head (BFsh), semitendinosus (ST), vastus lateralis (VL), rectus femoris (RF) and vastus medialis (VM), while no sex-specific differences were found for the semimembranosus (SM) and vastus intermedius (VI). However, female skiers had a proportionally larger SM and VI within the corresponding muscle group. Regarding strength, male skiers demonstrated a greater absolute and relative maximal voluntary torque during knee flexion and extension. However, all these studies were cross-sectional and/or of short duration, which limits the ability to identify growth-related changes in HAM morphology over time.

Therefore, the aim of this study was to investigate the longitudinal changes in the architecture and size of the HAM muscles, to verify the hypotheses of potential sex-specific differences in these adaptations, and their association with biological maturation.

## Materials and methods

### Study design, participants and ethics

The present study was designed as a cohort study in which baseline and 4-year follow-up measurements were conducted at the beginning of the ski season. The baseline measurements were conducted between November 2018 and February 2019, and the 4-year follow-up measurements between November 2022 and February 2023. Data analysis was performed between March 2023 and June 2023. From the original cross-sectional study by Fitze et al. [12], in which 95 youth competitive alpine skiers aged 15 years participated, 59 adolescent skiers aged 19 years (27 women and 33 men) could be recruited for the 4-year follow-up examination. Alpine skiers who were members of certified regional performance centres and represented the best skiers in their age group throughout Switzerland were eligible to participate. The exclusion criteria for the baseline assessment were as follows: skiers participating in a return-to-sport programme following an injury, and skiers with chronic or systemic diseases. During the four-year study period, all skiers participated in regular on-snow and off-snow training. However, no detailed training data was collected. In the period between baseline and 4-year follow-up, 5 female and 1 male participant suffered an ACL injury in the right knee and were therefore analysed separately. Table 1 provides an overview of the characteristics of the participants without ACL injury (w/o ACL injury, *n* = 53) and the participants with ACL injury (ACL injury, *n* = 6). The characteristics of the study participants at baseline and at the 4-year follow-up measurements can be seen in Table 1.

**Table 1.** Uninjured (*n* = 53) and ACL-injured (*n* = 6) study participant characteristics at baseline and at the 4-year follow-up.

Characteristic	Baseline		4-year follow-up	
**Uninjured**	**Female (*n* = 21)**	**Male (*n* = 32)**	**Female (*n* = 21)**	**Male (*n* = 32)**
Age (y)	14.8 ± 0.7	14.9 ± 0.6	18.8 ± 0.7	18.9 ± 0.6
Maturity offset (y)	2.3 ± 0.6	0.7 ± 0.8	–	–
Body height (cm)	164.1 ± 5.4	169.3 ± 7.7	167.6 ± 5.2	177.6 ± 5.7
Body mass (kg)	54.5 ± 6.2	59.1 ± 9.7	62.0 ± 6.9	76.2 ± 7.6
BMI	20.3 ± 2.0	20.5 ± 2.0	22.1 ± 2.1	24.1 ± 1.9
Femur length (cm)	36.9 ± 1.8	38.0 ± 1.9	38.0 ± 2.3	40.4 ± 1.7
**ACL-injured**	**Female (*n* = 5)**	**Male (*n* = 1)**	**Female (*n* = 5)**	**Male (*n* = 1)**
Age (y)	15.0 ± 0.7	14.6	19.0 + 0.7	18.6
Maturity offset (y)	2.4 ± 0.6	0.24	–	–
Body height (cm)	161.5 ± 6.6	171.0	163.8 ± 5.2	185.0
Body mass (kg)	56.9 ± 8.2	54.0	62.1 ± 7.5	80.0
BMI	21.7 ± 1.5	18.5	23.1 ± 1.7	23.4
Femur length (cm)	35.8 ± 1.5	40.0	36.7 ± 1.4	41.2

The data are presented as mean ± standard deviation.

ACL = anterior cruciate ligament, BMI = body mass index.

The study protocol was approved by the local ethics committee of the Canton of Zurich (KEK-ZH-NR: 2017–01395) and was in accordance with the ethical standards of the Declaration of Helsinki and the national laws of Switzerland. Written informed consent was obtained from all participants. For participants under the age of 14, consent was obtained from their legal guardians.

### Anthropometrics and maturity offset estimations

Body mass and height were measured using a body scale and a tape measure and body mass index (BMI) was calculated. Biological maturation was estimated using the non-invasive method developed by Mirwald et al. [14], which has been validated for the application in alpine ski racers [6]. The sex-specific Mirwald formula calculates the maturity offset considering the leg length (derived from body height and seat height) and the chronological age of the skier at the time of measurement. The maturity offset indicates the time difference between the measurement and the age of the peak growth rate. A negative maturity offset reflects the time before the skier is expected to reach the peak growth rate, while a positive value indicates the time that has elapsed since reaching the peak growth rate.

### Retrospective 4-year follow-up interviews on injury occurrence

At the follow-up examination, all skiers were retrospectively interviewed during a 15-min Microsoft Teams call to collect data on any ACL injuries that had occurred in the 4-year period between the baseline examination and the follow-up examination. ACL injuries had to be confirmed by a medical diagnosis to be counted as such.

### Ultrasound measurements

The ultrasound measurements were performed at the Swiss Centre for Musculoskeletal Imaging (SCMI) using the Aixplorer Ultimate ultrasound machine (SuperSonic Imagine, Aix-en-Provence, France). Two experienced examiners performed the ultrasound examinations (MVF at baseline and DPF at 4-year follow-up). Participants were instructed to lie prone on a massage table with the ankles positioned on the edge and the feet in a neutral position while the hip and knee joints were fully extended.

The protocols for acquiring and analysing ultrasound images have been described in detail in previous studies [12,15,16]. The intra-session reliability of ultrasound imaging for measuring HAM morphology has been shown to be high in these studies. Franchi and colleagues [15] reported ICC values of 0.96 (95% CI: 0.91–0.98) for the Lf and 0.84 (95% CI: 0.73–0.95) for the PA. Regarding ACSA, the study by Franchi et al. [16] reported ICC values of 0.97 (95% CI: 0.87–0.99) for the BFlh, 0.85 (95% CI: 0.36–0.98) for BFsh, 0.99 (95% CI: 0.93–0.99) for ST and 0.96 (95% CI: 0.74–0.99) for SM.

First, anatomical reference points, specifically the greater trochanter and the distal end of the lateral femoral condyle, were palpated and marked on the participants’ right leg. To determine the femur length, the distance between these reference points was measured with a tape measure. Additionally, marks were applied at 30%, 40%, 50% and 60% of this length. The medial and lateral borders of the BFlh were identified and marked using transversal ultrasound scans, which defined the acquisition path for the longitudinal panoramic scan.

**Figure 1.**
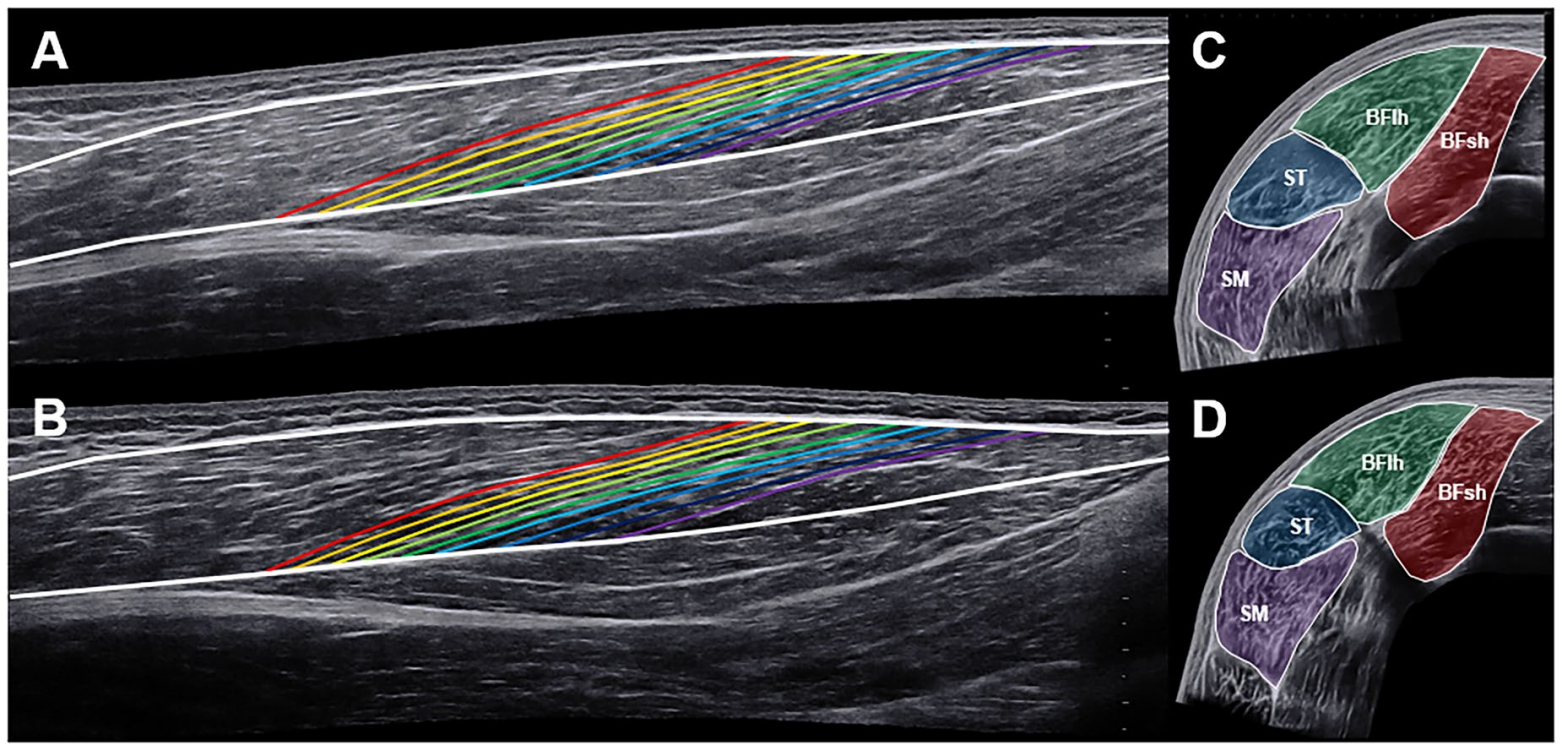
Representative ultrasound scans showing the biceps femoris long head muscle architecture at baseline (A), at the 4-year follow-up (B), as well as the hamstring anatomical cross-sectional areas at baseline (C) and at the 4-year follow-up (D). BFlh = biceps femoris long head, BFsh = biceps femoris short head, ST = semitendinosus, SM = semimembranosus.

The architecture of the BFlh muscle was measured with the linear SuperLinear SL18-5 transducer (SuperSonic Imagine, Aix-en-Provence, France). In panoramic mode, the ultrasound probe was moved from the distal to the proximal musculotendinous junction. Care was taken to move the transducer slowly and in a controlled manner to avoid excessive pressure on the underlying tissue. The orientation of the transducer was adjusted during imaging to ensure that the fascicles and superficial and intermediate aponeuroses remained clearly visible. Figure 1A and 1B shows representative images of baseline and 4-year follow-up measurements.

The HAM ACSAs were measured with the SuperLinear SL10-2 linear transducer (SuperSonic Imagine, Aix-en-Provence, France). The transducer was moved from lateral to medial in panoramic mode, rotating slightly during image acquisition to ensure continuous contact. Figure 1C and 1D shows representative images of baseline and 4-year follow-up measurements. In all measurements, ultrasound gel was applied along the acquisition path as a conductive medium and ensure smooth, consistent movement of the transducer.

### Ultrasound analysis

Image analysis was performed out by an experienced rater (DPF) *via* ImageJ software (National Institutes of Health, Bethesda, MD). For the analysis of muscle architecture, including quantification of Lf and PA, the superficial and intermediate aponeuroses were traced and nine fascicles (three from the proximal, middle and distal regions) were drawn. For statistical analysis, the median values of these measurements were then used. ACSAs were analysed in random order by tracing the contours of each HAM muscle (i.e. BFlh, BFsh, ST and SM) at each location relative to femur length (i.e. 30%, 40%, 50% and 60%). The largest ACSA from each of the four measurement sites was identified as ACSA_max_ and used for statistical analysis.

### Statistical analysis

The statistical analysis was performed in Python (version 3.11). Variance homogeneity was tested using the Levene test and the normal distribution of the data was tested using the Shapiro–Wilk test. Parametric tests were used for normally distributed data. If the Shapiro–Wilk test showed non-normality, but skewness and kurtosis were within the thresholds <2.0 and <7.0, respectively, the parametric tests were supplemented by bias-corrected and accelerated bootstrapping with 10’000 samples. The main analysis was performed on the uninjured skiers (*n* = 53) using a mixed ANOVA with time (baseline vs. 4-year follow-up) as the within-subjects factor and sex (female vs. male) as the between-subjects factor. A Bonferroni correction was applied to all ANOVA models to control for multiple comparisons. If a significant main effect or interaction effect was observed, paired (within sex) or unpaired (between sexes) *t* tests were performed as post hoc analyses and Cohen’s *d* was reported as the effect size. The classification of effect sizes followed the guidelines of Cohen [17]: *d* = 0.2 was considered a small effect, *d* = 0.5 indicated a medium effect, and *d* = 0.8 corresponded to a large effect. The significance level was set to *p* < 0.05. Participants who suffered an ACL injury during the study period (*n* = 6) were analysed descriptively. The associations between biological maturation and relative changes in BFlh Lf, BFlh PA, femur length and total HAM ACSA_max_ were examined using linear regression analyses. Statistical significance was set at *p* < 0.05.

## Results

The main and interaction effects of the mixed ANOVA for uninjured skiers are shown in Table 2. For the muscle architecture variable Lf, no significant effect of time (*F* = 1.96, *p* = 0.168, η^2^ = 0.037), sex (*F* = 0.94, *p* = 0.337, η^2^ = 0.018) or their interaction (*F* = 0.86, *p* = 0.358, η^2^ = 0.017) was found. For PA, a significant time effect (*F* = 12.48, *p* < 0.001, η^2^ = 0.197) and interaction effect (*F* = 5.54, *p* = 0.023, η^2^ = 0.098) were found, while there was no significant effect for sex (*F* = 0.49, *p* = 0.489, η^2^ = 0.009). For the muscle size variable ACSA_max_, significant effects of time (*F* = 35.58–125.74, all *p* < 0.001, η^2^ = 0.411–0.712), sex (*F* = 5.10–29.63, all *p* ≤ 0.028, η^2^ = 0.091–0.368) and their interaction (*F* = 7.17–29.14, all *p* < 0.010, η^2^ = 0.123–0.364) were found across all HAM muscles.

**Table 2.** Mixed ANOVA results for uninjured skiers (*n* = 53).

Variable	Effect	*F*	*p* value	η²
**Muscle architecture (BFlh)**				
Lf (cm)	Time	1.96	0.168	0.037
	Sex	0.94	0.337	0.018
	Time × sex	0.86	0.358	0.017
PA (°)	Time	12.48	<0.001	0.197
	Sex	0.49	0.489	0.009
	Time × sex	5.54	0.023	0.098
**Muscle size (ACSA_max_)**				
BFlh (cm^2^)	Time	125.74	<0.001	0.711
	Sex	11.29	0.002	0.181
	Time × sex	29.14	<0.001	0.364
BFsh (cm^2^)	Time	56.74	<0.001	0.527
	Sex	29.63	<0.001	0.368
	Time × sex	7.17	0.010	0.123
ST (cm^2^)	Time	35.58	<0.001	0.411
	Sex	15.52	<0.001	0.233
	Time × sex	17.92	<0.001	0.260
SM (cm^2^)	Time	98.4	<0.001	0.659
	Sex	5.10	0.028	0.091
	Time × sex	11.33	0.0015	0.182
HAM (cm^2^)	Time	126	<0.001	0.712
	Sex	22	<0.001	0.301
	Time × sex	25.66	<0.001	0.335

Lf = fascicle length, PA = pennation angle, ACSA_max_ = maximal anatomical cross-sectional area, BFlh = biceps femoris long head, BFsh = biceps femoris short head, ST = semitendinosus, SM = semimembranosus, HAM = hamstrings.

Table 3 shows the muscle architecture and size variables of the uninjured skiers at baseline and at the 4-year follow-up, as well as the results of the post hoc analyses. Over the course of 4 years, there was no significant change in Lf for both sexes (females: *p* = 0.125, *d* = 0.269, males: *p* = 0.618, *d* = 0.065). Male skiers showed a significant increase in PA (*p* < 0.001, *d* = 0.743), while it remained unchanged for female skiers (*p* = 0.639, *d* = 0.096). The ACSA_max_ of all HAM muscles increased significantly over time in both sexes (all *p* < 0.001, *d* = 0.453–1.804), except for the ST in female skiers, which remained unchanged (*p* = 0.499, *d* = 0.112). For both time points, no differences were found in Lf (baseline: *p* = 0.172, *d* = 0.386, follow-up: *p* = 0.608, *d* = 0.143) and PA (baseline: *p* = 0.549, *d* = 0.170, follow-up: *p* = 0.056, *d* = 0.522) between the two sexes. At baseline, male skiers displayed a significantly greater ACSA_max_ for the BFsh (*p* = 0.001, *d* = 0.913), while no sex-specific differences in ACSA_max_ were observed for the remaining HAM muscles (*p* = 0.074–0.642, *d* = 0.131–0.504). At follow-up, male skiers showed greater ACSA_max_ in all HAM muscles than females (all *p* < 0.001, *d* = 1.039–1.988).

**Table 3.** Biceps femoris long head muscle architecture and hamstrings maximal anatomical cross-sectional area at baseline and at the 4-year follow-up of uninjured skiers (*n* = 53).

	Baseline		4-year follow-up	
Variable	Female (*n* = 21)	Male (*n* = 32)	Female (*n* = 21)	Male (*n* = 32)
**Muscle architecture (BFlh)**				
Lf (cm)	8.6 ± 1.3	9.1 ± 1.3	9.0 ± 1.5	9.2 ± 1.6
PA (°)	11.9 ± 2.5	11.4 ± 2.5	12.1 ± 2.0	13.3 ± 2.6*
**Muscle size (ACSA_max_)**				
BFlh (cm^2^)	9.6 ± 1.9	10.2 ± 1.9	10.5 ± 2.0*	13.2 ± 1.8*#
BFsh (cm^2^)	5.6 ± 1.3	7.1 ± 1.9#	6.5 ± 1.1*	9.2 ± 1.7*#
ST (cm^2^)	8.5 ± 1.5	9.1 ± 1.8	8.7 ± 1.6	11.4 ± 1.8*#
SM (cm^2^)	9.8 ± 2.0	10.1 ± 2.1	11.3 ± 1.7*	13.2 ± 2.0*#
HAM (cm^2^)	33.6 ± 5.7	36.6 ± 6.2	36.9 ± 4.7*	47.1 ± 5.3*#

The data are presented as mean ± standard deviation.

Lf = fascicle length, PA = pennation angle, ACSA_max_ = maximal anatomical cross-sectional area, BFlh = biceps femoris long head, BFsh = biceps femoris short head, ST = semitendinosus, SM = semimembranosus, HAM = hamstrings.

Significant differences between time points within the same sex are indicated by * and significant differences between sexes at the same time point are indicated by #.

Table 4 shows descriptive HAM muscle architecture and size data for ACL-injured skiers.

**Table 4.** Biceps femoris long head muscle architecture and hamstrings maximal anatomical cross-sectional area at baseline and at the 4-year follow-up of anterior cruciate ligament-injured skiers (*n* = 6).

	Baseline		4-year follow-up	
Variable	Female (*n* = 5)	Male (*n* = 1)	Female (*n* = 5)	Male (*n* = 1)
**Muscle architecture (BFlh)**				
Lf (cm)	9.2 ± 1.0	8.0	9.0 ± 1.7	7.8
PA (°)	11.6 ± 1.4	9.0	11.2 ± 0.7	8.5
**Muscle size (ACSA_max_)**				
BFlh (cm^2^)	10.5 ± 1.8	11.1	12.3 ± 2.3	15.0
BFsh (cm^2^)	5.6 ± 1.1	6.5	6.7 ± 1.4	10.1
ST (cm^2^)	6.9 ± 1.8	3.6	4.9 ± 2.8	5.3
SM (cm^2^)	10.1 ± 1.6	11.3	11.9 ± 2.1	15.3
HAM (cm^2^)	32.9 ± 3.7	32.3	35.0 ± 5.3	45.7

The data are presented as mean ± standard deviation for female skiers and as single values for the male skier.

Lf = fascicle length, PA = pennation angle, ACSA_max_ = maximal anatomical cross-sectional area, BFlh = biceps femoris long head, BFsh = biceps femoris short head, ST = semitendinosus, SM = semimembranosus, HAM = hamstrings.

**Figure 2.**
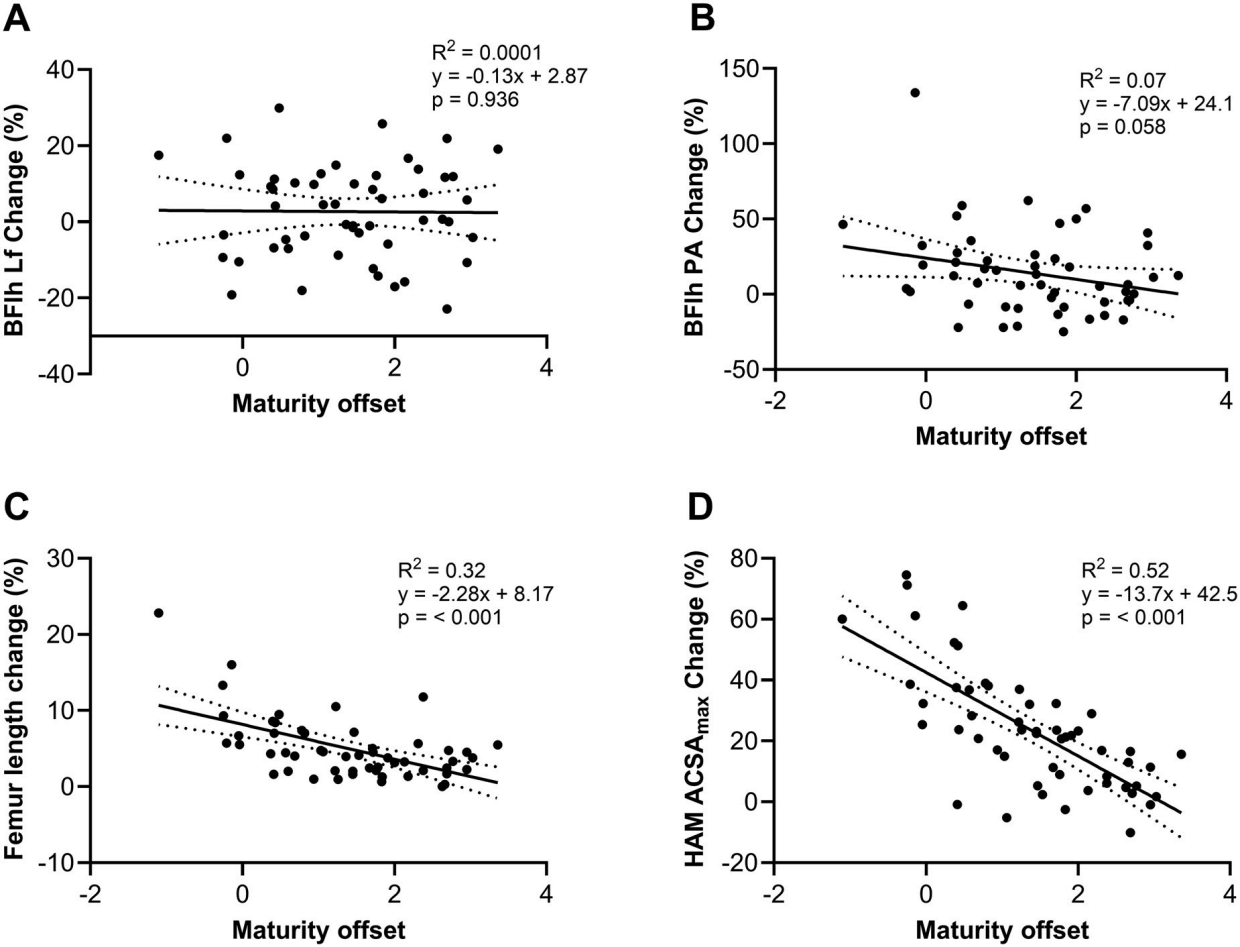
Linear regression analyses illustrating the associations between the maturity offset and the relative changes in biceps femoris long head fascicle length (A), biceps femoris long head pennation angle (B), femur length (C), and total hamstring maximal anatomical cross-sectional area (D). BFlh = biceps femoris long head, Lf = fascicle length, PA = pennation angle, HAM = hamstrings, ACSA_max_ = maximal anatomical cross-sectional area.

Figure 2 illustrates the associations between maturity offset and the relative changes in BFlh Lf (2 A), BFlh PA (Figure 2B), femur length (Figure 2C), and total HAM ACSA_max_ (Figure 2D). There was no significant association between maturity offset and the relative change in BFlh Lf (*R*^2^ = 0.0001, *p* = 0.936) or BFlh PA (*R*^2^ = 0.07, *p* = 0.058). Conversely, maturity offset was significantly associated with the relative change in femur length (*R*^2^ = 0.32, *p* < 0.001) and the total HAM ACSA_max_ (*R*^2^ = 0.52, *p* < 0.001).

## Discussion

The aim of this study was to investigate changes in HAM muscle architecture and size over a period of four years in female and male adolescent competitive alpine skiers and to examine their association with biological maturation. The most important findings were that the BFlh Lf did not change over time in either sex while the PA only increased in male skiers. Regarding HAM ACSA_max_, both sexes showed increases, with a greater increase in male skiers. As shown descriptively, female skiers who suffered an ACL injury during the study period displayed a lower ACSA_max_ of the ST at both the baseline examination and the follow-up compared to their uninjured counterparts. Maturity offset was negatively associated with femur length and total HAM ACSA_max_, indicating that the lower the biological maturation, the greater the growth potential for these variables.

No changes in BFlh Lf were observed for either sex. Previous studies regarding Lf have reported significant differences in Lf between boys and men but only small to moderate differences between boys aged 14 and 16 years [10,18–20]. Furthermore, Kubo and colleagues [21] demonstrated a difference in Lf between boys and 15-year-old adolescents but not between adolescents and adults. Given the mean age of the skiers in the present study (14.8 ± 0.7 years for females and 14.9 ± 0.6 years for males), it could be argued that they may have already reached adult-level Lf. Although no changes in BFlh Lf were observed in either females or males, femur length increased in both sexes. As a result, the Lf relative to the femur length decreased over time, which may have functional consequences. These findings could suggest that BFlh Lf reaches its adult length before bone growth is complete. Although we did not measure the entire BFlh muscle–tendon unit length over time, it could be speculated that after the Lf reaches its adult level, any subsequent elongation of the muscle–tendon unit is due to tendon lengthening. In addition to growth, resistance training involving lengthening muscle actions [22] can increase Lf. Although no detailed training data was collected, it may be speculated that the overall training stimulus to which the skiers were exposed during this development phase was insufficient to cause such adaptations in BFlh.

Regarding BFlh PA, only male skiers showed an increase, despite both sexes showing an increase in BFlh ACSA_max_. PA is known to increase in response to muscle hypertrophy [23], serving as a packing strategy to increase the physiological CSA, which is the CSA perpendicular to the fascicle orientation, within a given muscle volume [24]. During maturation, changes in PAs have been shown to be muscle- and site-specific [10,18,20]. A plausible explanation for these sex-specific paths of the PA may be attributed to the lower biological maturation status of male skiers. As a result, males presented greater potential for increases in PA during the period following the baseline measurements. For female skiers who suffered an ACL injury, no descriptive changes in either Lf or PA were observed. Although speculative, this may indicate that muscle architecture in the BFlh is less sensitive to injury-related factors, or it could suggest that the rehabilitation strategies used did not significantly alter muscle architecture over the observed period.

The results revealed sex differences in HAM ACSA_max_ at the 4-year follow-up, where males showed greater ACSA_max_ values across all HAM muscles (BFlh, BFsh, ST, SM) than females. Our findings at the follow-up are consistent with a previous study from our research group [13], which reported similar sex-specific differences in HAM and quadriceps muscle size among elite ski racers. It is well documented that female athletes, on average, display lower muscle mass than their male counterparts [25,26]. Both sexes showed increases in HAM ACSA_max_ over time, which highlights the responsiveness of the HAM to growth and physical training during this period. However, the lack of a change in ST ACSA_max_ in females suggests potential muscle- and sex-specific differences in the hypertrophic response. Descriptively, female skiers with ACL injuries showed an increase in SM ACSA_max_, while there was no change for BFlh, BFsh, ST, or total HAM ACSA_max_. The distal ST tendon is commonly harvested as autologous graft tissue for ACL reconstruction, and it is well documented that this procedure leads to a reduction in the ST ACSA and muscle volume [27–29]. Therefore, the observed increase in SM ACSA_max_ could represent a compensatory response, which is consistent with the results of previous studies in patients and recreational athletes following unilateral ACL reconstruction [27,28].

Based on these observations, our findings may have important implications for ACL injury mechanisms. Both the SM and ST are part of the medial HAM and act as internal rotators of the tibia, while the BFlh, as part of the lateral HAM, has an external rotatory function [30]. During ski-specific movements, excessive internal rotation of the tibia in relation to the femur has been identified as a common mechanism leading to ACL injuries [4,31]. The concurrent decrease in ST and increase in SM observed in females who sustained an ACL injury could therefore represent an unfavourable shift within the medial HAM, leading to a dominance of internal rotation forces. This imbalance could be further accentuated if the lateral HAM are relatively small and/or weak. This interpretation is supported by the findings of our previous research. In a study with adolescent competitive alpine skiers [12], we showed that a larger BFlh ACSA_avg_ was associated with a reduced risk of sustaining a traumatic lower extremity injury in the following season. Furthermore, in a study with elite alpine skiers [13] we found that female skiers displayed a proportionally larger SM within the HAM muscle group compared to males. Since the SM acts as an internal rotator of the tibia, this structural characteristic may predispose female athletes to greater internal rotation moments during HAM activation. The present study extends these findings by showing that, following ACL injury and reconstruction, this imbalance may be further increased by compensatory SM hypertrophy and persistent ST reduction, potentially increasing ACL loading and the risk of re-injury.

Another important finding of this study is the significant associations between maturity offset and the relative changes in femur length and total HAM ACSA_max_. The maturity offset explained a significant portion of the variability in both femur length (32%) and total HAM ACSA_max_ (52%) changes. These results support the concept that maturity status is a critical determinant of musculoskeletal development, with earlier-maturing individuals likely to experience more pronounced increases in both bone length and muscle size. In contrast, there was no association between maturity offset and relative changes in BFlh Lf or PA. These findings suggest that while overall bone length and muscle growth and bone are influenced by maturity status, specific architectural adaptations of individual muscles, such as Lf and PA, may be driven by other factors.

The results of this study may be important for training, injury prevention and rehabilitation of adolescent competitive alpine skiers. Based on the greater developments in HAM muscle size and BFlh PA in male skiers, resistance training programmes for female skiers may need to be adapted or intensified, as they are at higher risk of ACL injury during this phase of development [32]. The associations found between maturity offset and changes in femur length and total HAM ACSA_max_ also suggest that biological maturation should be considered when designing training interventions. Furthermore, training interventions should not only aim to develop total HAM muscle size, but also focus on the ratio of medial to lateral HAM. Specific exercises to develop the lateral HAM could have a preventive effect on excessive internal tibial rotation forces.

This study has several limitations that must be considered when interpreting the data. First, the sample size was relatively small, especially in the ACL-injured group, which limits the generalisability of the results. Second, the baseline and follow-up ultrasound measurements were performed by two different operators. Although a standardized protocol was followed on both occasions, the results may have been influenced by inter-operator variability. Third, due to time constraints, we focused only on measuring the HAM and did not measure the knee extensor muscles, although these may also play an important role in knee stabilisation and injury prevention. Fourth, no detailed training histories (type, frequency, duration and intensity) were collected during the study. The lack of this information limits the interpretation of the muscle architecture and size data. Future research should aim to include larger samples, longitudinal monitoring of both hamstring and quadriceps muscles, and continuous documentation of training variables to enable a more holistic understanding of muscle development and potential injury mechanisms during adolescence.

## Conclusions

The present study provides novel insights into the longitudinal changes in HAM muscle architecture and size in adolescent competitive alpine skiers. It emphasizes the critical role of biological maturation in determining musculoskeletal development. Our findings revealed that while both male and female skiers showed increases in HAM ACSA_max_ over the period of 4 years, males demonstrated greater hypertrophy across all HAM muscles. Descriptively, ACL-injured female skiers displayed smaller ST ACSA_max_ at both timepoints, emphasising the need for specific interventions. The significant associations between maturity offset and both femur length and total HAM ACSA_max_ highlight the importance of considering biological maturation when designing training and injury prevention programs for adolescent athletes.

## Data Availability

The datasets presented in this article are not readily available because their access is restricted to protect the interests of the project partner Swiss-Ski and their athletes. Requests to access the datasets should be directed to joerg.spoerri@balgrist.ch.
